# Editorial: Weed identification and integrated control

**DOI:** 10.3389/fpls.2023.1351481

**Published:** 2023-12-13

**Authors:** Pei Wang, Gerassimos Peteinatos, Aspasia Efthimiadou, Wei Ma

**Affiliations:** ^1^College of Engineering and Technology, Key Laboratory of Agricultural Equipment for Hilly and Mountain Areas, Southwest University, Chongqing, China; ^2^Soil and Water Resources Institute, Hellenic Agricultural Organization – ELGO, Athens, Greece; ^3^Institute of Urban Agriculture, Chinese Academy of Agricultural Sciences, Chengdu, China

**Keywords:** weed identification, integrated control, data resource, artificial intelligence, mechanical weed control

Weeds are among the major factors that could harm crop yield. Herbicides are commonly used for weed control but have drawbacks such as drift, runoff, crop injury, pollution, and herbicide resistance. Therefore, the development of integrated systems, incorporating mechanical weeding, rotation, variable spraying, and allelopathy inhibition, is crucial to enhance weed control strategies. Furthermore, precise weed control strategies hinge on the identification of individual weed specimens and their unique biophysical properties, enabling tailored approaches to effectively suppress each weed species.

Sensing technology such as 3D cameras, multispectral imaging, plays a pivotal role in providing precise spatial and temporal data on weeds in agricultural fields, including species, density, and growth stages (Peteinatos et al., 2014). Sensor techniques are also useful for recognizing, monitoring, and evaluating stresses on weeds or crops. Artificial intelligence technologies, especially deep learning, have excellent application prospects in agriculture (Bannerjee et al., 2018). Through deep learning, the establishment of a dataset of weeds can realize high-precision recognition of weeds in the field, which greatly improves the ability to distinguish between crops and weeds in the field (Rai et al., 2023). In addition, artificial intelligence technology combined with imaging technology provides real-time and efficient weed monitoring in the field, detecting and responding to weed problems in a timely manner. The combination of multi-sensor technology and artificial intelligence can customize effective weed control strategies based on precise spatial and temporal information about weeds in the field, minimizing herbicides use, reducing costs and protecting the environment (Wang et al., 2019).

This Research Topic contains four original articles from weed dataset creation to identification and precise control in the field.

An open access weed image dataset was created by Wang et al. Weed control plays a crucial role in determining crop yields. Precise identification of weed species is essential for automating weeding processes, such as applying suitable herbicides, determining hoeing positions, adjusting hoeing depth for specific plants, and minimizing crop damage. Deep learning techniques are extremely good at precisely identifying weeds and customizing weed control strategies. However, the lack of comprehensive weed datasets has hindered the application of advanced deep learning techniques in weed management. This study introduces a new dataset, Weed25, comprising 14,035 images representing 25 different weed species found in agricultural fields. The dataset includes images of both monocot and dicot weeds at various growth stages. To assess the dataset’s utility, popular deep learning models YOLOv3, YOLOv5, and Faster R-CNN were employed for training weed identification models. The results demonstrated that these models achieved average detection accuracies of 91.8%, 92.4%, and 92.15%, respectively, under the same training parameters. This suggests that Weed25 has the potential to serve as an effective resource for training real-time weed identification models in agricultural settings. The dataset is available at https://pan.baidu.com/s/1rnUoDm7IxxmX1n1LmtXNXw; the password is rn5h.

Xu et al. presented a multi-modal and multi-view image dataset for weed detection in open wheat fields. The dataset includes an RGB image, a depth image of the same scene and PHA images obtained through re-encoding depth images, providing three-dimensional structure features for weed detection in wheat fields. Additionally, the dataset contains images from nine different views, which offer a more comprehensive feature space for weed detection under complex backgrounds such as leaf occlusion and overlapping. The data were collected during experiments conducted in wheat fields, where weeds were allowed to grow naturally. The dataset includes images of different weed species commonly found in wheat fields. The dataset is valuable for promoting the development of weed detection methods in wheat fields, especially in addressing the challenges posed by grass weeds and complex backgrounds. The dataset is publicly available and can be accessed for further research and development of weed detection algorithms.

In the study of Zhu et al., the importance of weed control in maize fields and discusses the limitations of current weed control methods was emphasized. It also highlights the advantages of laser weeding, such as high efficiency, precise positioning, low seedling injury rate, and environmental friendliness. The introduction of machine vision and robotics in weed control has improved efficiency, reduced labor intensity, and minimized the use of herbicides. The paper provides an overview of previous studies on weed control robots and highlights the significance of online weed recognition and classification for accurate and targeted weeding. The study designed a YOLOX convolutional neural network-based weeding robot including a tracked mobile platform module, a weed identification module, and a robotic arm laser emitter module. Five-degree-of-freedom robotic arm designed according to the actual weeding operation requirements to achieve precise alignment of the laser. When the robot is in operation, it uses the texture and shape of the plants to differentiate between weeds and corn seedlings. The robot then uses monocular ranging to calculate the coordinates of the weeds using the triangle similarity principle, and it controls the end actuator of the robotic arm to emit the laser to kill the weeds. The paper concludes by discussing the potential applications and future research directions of the YOLOX-based blue laser weeding robot.

Finally, a second precision mechanical-chemical integrated weed control system was designed by Fang et al. The authors explored the impact of mechanical-chemical synergy as an alternative weeding practice on maize growth and yield. The study conducted experiments using different chemical reduction ratios and application methods, and measured growth characteristics such as leaf area and dry matter weight at the filling and maturity stages. The results showed that the leaf area of the mechanical-chemical synergistic treatments was larger than single mechanical or chemical weeding treatments at the filling stage. The dry matter weight of the mechanical-chemical weeding treatments was greater compared to the chemical weeding treatment at both the filling and maturity stages. The number of grains per ear, 1000 grain weight, and yield of the mechanical-chemical synergistic treatment were better than those of the chemical treatment. The study recommends a 50% chemical reduction ratio as the best option for reducing herbicide application without significantly affecting crop growth and yield.

## Author contributions

PW: Writing – original draft, Writing – review & editing. GP: Writing – original draft, Writing – review & editing. AE: Writing – original draft, Writing – review & editing. WM: Writing – original draft, Writing – review & editing.
